# A Novel Fluorescent Probe for Selective Detection of Hydrazine and Its Application in Imaging

**DOI:** 10.3390/bios11050130

**Published:** 2021-04-22

**Authors:** Ruo-Jun Man, Meng-Ke Wu, Bing Yang, Yu-Shun Yang

**Affiliations:** 1 Guangxi Biological Polysaccharide Separation, Purification and Modification Research Platform, Guangxi University for Nationalities, Nanning 530006, China; wmk135076@163.com; 2 School of Chemistry and Chemical Engineering, Nantong University, Nantong 226019, China; 3 Research Centre of Sensors and Functional Materials, Hi-Techjig Co. Ltd., Zhenjiang 212415, China

**Keywords:** hydrazine detection, fluorescent probe, biological imaging, thiazepine moiety, water samples

## Abstract

In this work, a novel fluorescent probe with first-time-selected thiazepine backbone, TZPzine-1, was developed for selective detection of hydrazine in water samples and living cells. Chosen from our recent anti-cancer agents, TZPzine-1 inferred structurally based advantages of the optical adjustability and the hydrazine-trapping approach. It also showed applicable properties including high sensitivity (LOD = 50 nM), wide linear range (0–15 equiv.), high selectivity (especially from competing species), rapid response (within 20 min), and practical steadiness in various pH (6.0–11.0) and temperature (15–50 °C) conditions. To satisfy the interdisciplinary requirements in environmental toxicology, TZPzine-1 was successfully applied in water samples and living cells. We hope that the information in this work, as well as the concept of monitoring the nitrogen cycle, may be referable for future research on systematic management.

## 1. Introduction

Nitrogen-based compounds, as components of the nitrogen cycle, travel all through the manufacturing industry, the environment, plants, animals, and humans [1]. Among them, due to its reductivity and basicity, hydrazine (NH_2_NH_2_) seems to be one of the most important species in industrial and medicinal applications [2,3]. This highly toxic hydrazine is common in wastewater from dyeing, synthetic, or zymotic factories [4,5]. Accordingly, its minimum limit of potential carcinogen was determined as 10 ppb by the US Environmental Protection Agency (EPA) [6]. Therefore, it is of significant value to monitor the level of hydrazine with novel methods.

Previous approaches for the detection of hydrazine include electrochemistry [7], Raman spectroscopy [8], colorimetry [9], chemiluminescence [10], spectrophotometry [11], and titrimetry [12]. To avoid a complex preparation and strict procedures, researchers who are interested in convenient detection have paid attention to fluorescent probes [13]. As reported, a typical probe includes the fluorophore (such as benzothiazole [14], 1,8-naphthalene imide [15], coumarin [16], rhodamine [17], phenothiazine [18], carbazole [19], and cyanine dye [20]) and the recognition group (such as acetyl [21], levulinate [22], 4-bromo butyrate [23], phthalimide [24], aldehyde [25], cyano [26]). As we claimed in our previous work [27], compared with complex tools (such as rare earth [28], aggregation-induced enhancement (AIE) [29], nanoparticles [30], or carbon dots [31]), small molecular probes were preferred in the closed-loop monitoring of hydrazine circulation. Thus, novel mechanisms for recognizing hydrazine with specific cases are still urgently needed.

**Figure 1:**
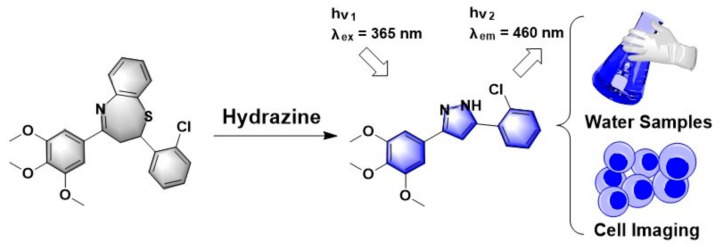
Illustration of the TZPzine-1 for the detection of hydrazine.

In this work, we introduced a novel structure which was obtained in our investigation of anti-cancer agents [32] for the detection of hydrazine in water samples and living cells. As shown in Figure 1, this thiazepine-containing probe, TZPzine-1 (“TZP” for thiazepine and “zine” for hydrazine), was transformed into pyrazole derivative by hydrazine under aerobic environment. Since the optimization of the substitutes and the oxidizing conditions might be another methodological story, here we focused on the detecting capability, with TZPzine-1 as an example. Structurally, we could conclude that TZPzine-1 had two potential advantages. One was that the optical properties could be regulated by changing the substitutes, while the other was that hydrazine was trapped in the fluorophore after the detection, unlike the departure in common mechanisms [21,22,23,24]. As compared with the previous reports in Table S1 in Supporting Information (SI), TZPzine-1 showed a fast response within 20 min and a high selectivity. The limit of detection (LOD) was calculated as 50 nM, fulfilling the EPA standard. We then performed the application of TZPzine-1 in water samples and living cells.

## 2. Experimental

### 2.1. Materials and Methods

The purchased chemicals were used directly without further purification. ^1^H NMR and ^13^C NMR spectra were recorded on a Bruker DRX-600 spectrometer (Karlsruhe, Germany), and the data were analyzed with MestreNova software (Santiago de Compostela, Spain). Mass spectra were recorded on Agilent 6540 UHD Accurate Mass Q-TOF LC/MS (Santa Clara, CA, USA). The UV-vis test was conducted on a Shimadzu UV-2550 spectrometer (Kyoto, Japan). All the fluorescence tests were performed on a Hitachi F-7000 Fluorescence Spectrometer (Tokyo, Japan). The imaging was conducted on a Leica TCS SP8 MP two-photon confocal fluorescent microscope (Weztlar, Germany).

The stocking solution of the probe TZPzine-1 was prepared in DMSO at the concentration of 1.0 mM. Other concentrations were obtained by dilution. In the selectivity experiments, the concentration of hydrazine was set as 300 μM, while that of the other analytes was set as 1.0 mM. The excitation wavelength was set at 365 nm. Both excitation and emission slit widths were 10 nm. The photomultiplier voltage was 600 V.

### 2.2. Water Sample Pretreatment

Water samples were collected from Guangxi University for Nationalities, Nanning (rainwater, tap water, and rice water), Zhenjiang Gexian Lake (lake water), Yangtze River Zhenjiang Section (river water), and the East China Sea near Nantong, Jiangsu Province, China (sea water). The samples were all coded and transported to our laboratory. Subsequently, the water samples were placed statically overnight and filtered. The pH values of the samples were tested to ensure that they were all between 7.0 and 8.0. The pretreated water samples were used as the solution system in the tests, instead of PBS, without further dilution.

### 2.3. Synthesis of the Probe TZPzine-1

**Figure 2:**
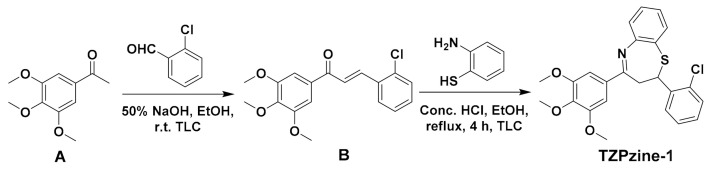
General synthesis route of the probe TZPzine-1.

The synthesis of the probe TZPzine-1 was illustrated in Figure 2. According to our previous report [32], 1-(3,4,5-trimethoxyphenyl) ethan-1-one (1 mmol, 0.210 g) was added to a 5 mL ethanol solution of 2-chlorobenzaldehyde (1 mmol, 0.140 g). Then, 50% NaOH (0.5 mL) was added to the mixture dropwise. The completion of the reaction was checked by thin layer chromatography. Afterwards, the mixture was filtered, washed by cold ethanol, and dried, to acquire the intermediate B (0.293 g, yielding 88.3%). Subsequently, intermediate B (0.5 mmol, 0.166 g) was dissolved in ethanol under reflux condition. To the hot solution, 2-aminothiophenol (0.5 mmol, 0.063 g) was added, and then concentrated hydrochloric acid was added dropwise. The reaction was kept running continuously for 4 h. The crude product was filtered, washed with cold ethanol, dried, and recrystallized to obtain the probe TZPzine-1 (0.141 g, yielding 64.2%). The grey-white powder melt point is 139–140 °C. ^1^H NMR (600 MHz, DMSO-*d*_6_) δ 7.65 (d, *J* = 7.68 Hz, 1H, ArH), 7.63 (d, *J* = 7.68 Hz, 1H, ArH), 7.55 (t, *J* = 7.7 Hz, 1H, ArH), 7.50 (d, *J* = 7.7 Hz, 1H, ArH), 7.46 (s, 2H, ArH), 7.38–7.32 (m, 2H, ArH), 7.30 (d, *J* = 7.9 Hz, 1H, ArH), 7.21 (t, *J* = 7.6 Hz, 1H, ArH), 5.48 (dd, *J* = 13.0, 4.6 Hz, 1H, -CH_2_-), 3.88 (s, 6H, (-OCH_3_)_2_), 3.77 (s, 3H, -OCH3), 3.55 (dd, *J* = 13.1, 4.6 Hz, 1H, -CH_2_-), 2.77 (t, *J* = 13.1 Hz, 1H, -S-CH-). ^13^C NMR (151 MHz, DMSO-*d*_6_) δ 168.28, 153.46, 152.67, 141.27, 140.90, 135.39, 132.58, 130.99, 130.70, 129.87, 129.77, 128.36, 128.15, 125.89, 125.65, 122.05, 105.24, 60.65, 60.23, 56.47. HRMS (ESI-TOF) m/z: [M + H]^+^ Calcd. for C_24_H_23_ClNO_3_S 440.1087, Found 440.1082.

## 3. Results and Discussion

### 3.1. Synthesis of the Probe TZPzine-1

The probe TZPzine-1 was synthesized according to the general route in Figure 2, as mentioned in our previous report in medicinal chemistry [32]. The structure was confirmed by satisfactory spectroscopic data (^1^H NMR, ^13^C NMR and HRMS, Figures S6–S9, SI). In this work, we only took TZPzine-1 as a representative, and the optical capability could actually be regulated by changing the substitutes.

### 3.2. Fluorescent Properties for Detecting Hydrazine

Under the excitation wavelength of 365 nm, the fluorescence spectra of the probe TZPzine-1 and the detecting system suggested that the weak emission peak of TZPzine-1 at 515 nm and the strong emission peak at 460 nm appeared after the addition of hydrazine. The response to hydrazine caused an over 30-fold enhancement in fluorescence intensity at 460 nm, and a Stokes shift of 95 nm. The fluorescence intensity came to the plateau when the concentration of the added hydrazine reached 30 equivalent (equiv. or Eq). Therefore, the sensing system consisting of TZPzine-1 (10 μM) and hydrazine (300 μM), in a PBS buffer (pH 7.4, 10 mM, 1% DMSO *v*/*v*), at 37 °C was used to check responses to different external conditions. Accordingly, the fluorescence signal of TZPzine-1 remained steady within the pH range of 6.0–12.0 while the detecting system was stable within 5.0–11.0, which inferred the wide window of 6.0–11.0 for practical use (Figure S1, SI). The test, under various temperature conditions, suggested that the probe and the detecting system were both steady in the range of 15–50 °C (Figure S2, SI), which was beneficial for the consistency between the environmental (usually 25 °C) and biological (usually 37 °C) samples. Moreover, the detecting procedure could be completed within 20 min, which was relatively rapid, whereas the response period could be extended to over 60 min when we used a nitrogen-filled balloon to set a simple hypoxic condition (Figure S3, SI). This might be an interesting point in the research of water eutrophication, but is not the focus of this work.

**Figure 3:**
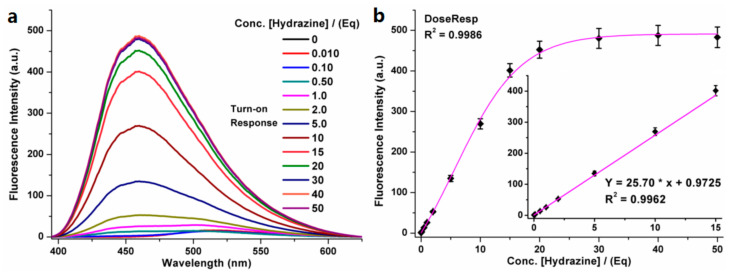
(**a**) The fluorescence spectra of TZPzine-1 (10 µM) in PBS buffer (10 mM, pH 7.4, 1% DMSO *v*/*v*) after treatment with hydrazine (0–500 µM) for 20 min; (**b**) The curve between the fluorescence intensity and the concentration of hydrazine (0–500 µM); b-Inner: The linear relationship between the fluorescence intensity and the concentration of hydrazine (0–150 µM).

As shown in Figure 3, an obvious turn-on variation in the fluorescence at 460 nm could be observed from the fluorescent spectrum of TZPzine-1 (10 μM), with increasing concentration of hydrazine (0–500 µM). As we mentioned, the plateau was reached with 30 equiv. hydrazine. Accordingly, the linear range was 0–15 equiv. (0–150 µM). The limit of detection (LOD) was calculated as 50 nM (0.005 equiv.) from the formula 3.29 σ/k (σ meant the deviation of 25 tests in the detecting system with no hydrazine; k meant the slope of the linear fitting). The limit of quantity (LOQ) was tested to be 100 nM (0.010 equiv.) by continuously diluting the concentration of hydrazine. The wide linear range and the high sensitivity guaranteed the capability of TZPzine-1 in practical applications.

### 3.3. Selectivity for Hydrazine

**Figure 4:**
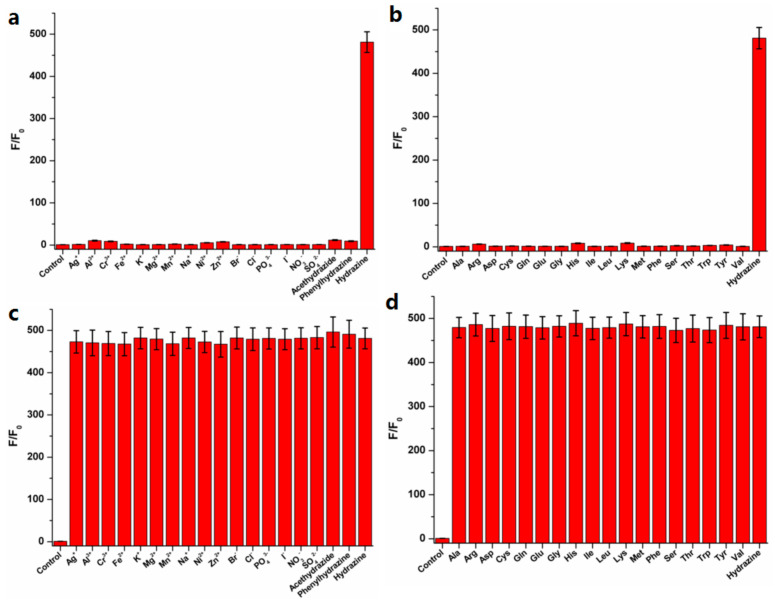
The intensity ratios (F/F_0_) at 460 nm in PBS buffer (10 mM, pH 7.4, 1% DMSO *v*/*v*) at 37 °C indicated the selectivity of TZPzine-1 towards hydrazine from (**a**) ions or competing species, (**b**) amino acids, as well as in the coexistence systems containing (**c**) ions or competing species, (**d**) amino acids.

Subsequently, based on the intensity ratios (F/F_0_, F: Intensity of response; F_0_: Intensity of control) at 460 nm, the selectivity of TZPzine-1 for hydrazine was checked from various analytes (Figure 4a,b). As shown, except for hydrazine, none of the analytes could cause an obvious increase in the fluorescent signal. In particular, the competing species acetohydrazide and phenylhydrazine could be easily distinguished from hydrazine. This might be quite important in the detection of hydrazine. Meanwhile, in the coexistence system with both hydrazine and other analytes, the detection of hydrazine was not interfered (Figure 4c,d). Accordingly, the selectivity of TZPzine-1 for hydrazine was steady in both situations of independence and coexistence, which further ensured the potential of this probe.

### 3.4. The Reaction Mechanism

**Figure 5:**
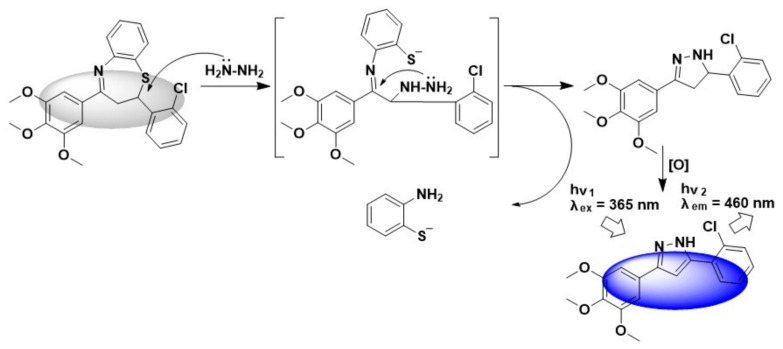
The illustrated reaction mechanism between TZPzine-1 and hydrazine.

The reaction mechanism between TZPzine-1 and hydrazine, as illustrated in Figure 5, was different from typical ones. Since the theoretical mechanism involved a slight oxidation after the cyclization, it was quite difficult to separate the product from the detecting system due to the stickiness. Still, we were able to check the HRMS spectrum of the pyrazole product (Figure S9, SI) with the help of the reports on similar backbones [33,34]. In this mechanism, hydrazine was trapped in the fluorophore after the detection, unlike the departure in common ones. Moreover, this mechanism agreed with the selectivity of TZPzine-1 for hydrazine from competing species such as acetohydrazide and phenylhydrazine according to the factors, including electronic status, steric hindrance, and product stability. The Job’s plot analysis for TZPzine-1 and hydrazine interaction (Figure S4, SI) confirmed the 1:1 ratio of binding pattern, which agreed with the trapping one. Since the probe in this work chose a trapping mechanism, although the highly toxic hydrazine was better treated (trapped into the detecting product), the detected product could not be easily transformed into the synthetic precursor of the probe. Accordingly, the downstream use of the detecting product was realized by including it as the intermediate in medicinal chemistry [33,34]. Actually, the detection of hydrazine required less raw materials than the medicinal development. Therefore, although a bit different from the typical reversibility of previous probes [35,36], the recycling of the probe in this work was achieved within a systemic circulation of several industrial chains.

### 3.5. Application in Water Samples

Since hydrazine is a toxic pollutant mainly found in wastewater, we used water samples from different sources to check the performance of TPZzine-1. The water samples were collected in the cities of Nanning, Zhenjiang, and Nantong in China. They covered the sources of rain, tap, rice, lake, river, and sea. As seen in Figure S5, SI, in the detecting systems with different water samples, TZPzine-1 could successfully monitor the concentration of hydrazine with no obvious interference. This result was beneficial for the concept of close-loop detection of the nitrogen cycle.

### 3.6. Cell Imaging

**Figure 6:**
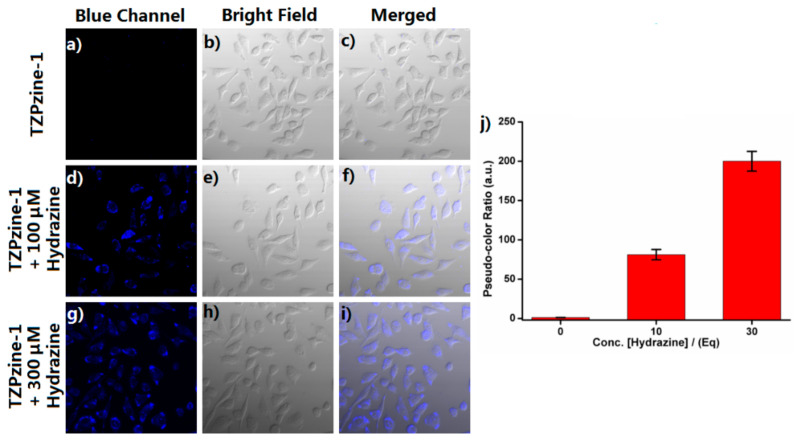
The confocol images of living HeLa cells. Further incubations for 20 min with (**a**–**c**) PBS, (**d**–**f**) 100 μM hydrazine, and (**g**–**i**) 300 μM hydrazine were conducted, respectively, after a pre-incubation with 10 μM TZPzine-1 for 30 min. λ_ex_ = 365 nm, blue channel: 400–490 nm. (**j**) The pseudo-color ratio calculated from three random patches in blue channel pictures of each concentration.

As an anti-cancer candidate, TZPzine-1 itself showed anti-proliferation activity. Fortunately, the detecting period was in 20 min and TZPzine-1 could easily be degraded in vivo, thus guaranteeing the safety of the detecting system. Afterwards, we conducted biological imaging with TZPzine-1 in living HeLa cells (Figure 6). After being co-cultured with TZPzine-1 only for 30 min, the cells indicated no obvious intracellular fluorescence, which ensured the low background noise, as shown in Figure 6a–c. When the cells were treated with TZPzine-1 for 30 min and then incubated with hydrazine of 100 μM (Figure 6d–f) and 300 μM (Figure 6g–i) for 20 min, respectively, the enhancement of the fluorescence signal in the blue channel could be observed. Remarkably, a higher concentration of hydrazine caused larger fluorescence enhancement (Figure 6j). Consequently, the results inferred the applicative potential of TZPzine-1 in cellular imaging. Therefore, we could generate the connection of environmental toxicology by checking the data in water and cellular samples.

## 4. Conclusions

In summation, we built a novel fluorescent probe with first-time-selected thiazepine backbone, TZPzine-1, for the detection of hydrazine in water samples and living cells. It was picked up in our recent investigation of anti-cancer agents. TZPzine-1 inferred potential advantages including the optical adjustability and the hydrazine-trapping approach. Compared with the typical probes for hydrazine in previous reports (Table S1, SI), TZPzine-1 indicated the necessary advantages including high sensitivity (LOD = 50 nM), wide linear range (0–15 equiv.), high selectivity (especially from competing species), rapid response (within 20 min), and practical steadiness in various pH (6.0–11.0) and temperature (15–50 °C) conditions. Whilst fulfilling the EPA standard, we successfully applied TZPzine-1 in the detection of hydrazine in water samples and living cells. This kind of interdisciplinary detection might satisfy the requirements in environmental toxicology, which also agrees with the concept of monitoring the nitrogen cycle. We hope that the information in this work may be favorable for systematic management in future investigations.

## Supplementary Materials

The supporting information is available online at https://www.mdpi.com/article/10.3390/bios11050130/s1. Figure S1: The fluorescence intensity at 460 nm changes in various pH environments, Figure S2: The fluorescence intensity at 460 nm changes in various temperature conditions, Figure S3: The fluorescence intensity at 460 nm changes in various time conditions, Figure S4: Job’s plots analysis for TZPzine-1 and hydrazine interaction, Figure S5: The application of TZPzine-1 in detecting systems with different water samples from rain, tap, rice, lake, river and sea, Figures S6–S9: NMR and HRMS spectra, Table S1: The comparison with typical probes for hydrazine.
