# Supplementary material for: A Novel Fluorescent Probe for Selective Detection of Hydrazine and Its Application in Imaging
Source: Biosensors (Basel). 2021 Apr 22;11(5):130. doi: 10.3390/bios11050130 (PMC8143562; doi:10.3390/bios11050130)
Supplement: Supplementary file 1 [file biosensors-11-00130-s001.zip › biosensors-1161912-supplementary.pdf]

Supporting Information

A Novel Fluorescent Probe for Selective Detection of Hydrazine and Its Application in Imaging

Ruo-Jun Man [1,*,†], Meng-Ke Wu [1,†], Bing Yang [2,*] and Yu-Shun Yang [3,*]

[1] Guangxi Biological Polysaccharide Separation, Purification and Modification Research Platform, Guangxi University for Nationalities, Nanning 530006, China;wmk135076@163.com
[2] School of Chemistry and Chemical Engineering, Nantong University, Nantong 226019, China
[3] Research Centre of Sensors and Functional Materials, Hi-Techjig Co. Ltd., Zhenjiang 212415, China
[*] Correspondence: manruojun@163.com (R.-J.M.); yangbing111@ntu.edu.cn (B.Y.); ys_yang@nju.edu.cn (Y.-S.Y.); Tel.:+86-25-89682572 (Y.-S.Y.)
[†] The authors contributed equally to this work.

Table of Contents

The fluorescence intensity at 460 nm changes in various pH environments.. ....S1

The fluorescence intensity at 460 nm changes in various temperature conditions. S2

The fluorescence intensity at 460 nm changes in various time conditions S3

Job's plots analysis for **TZPzine-1** and hydrazine interaction....................................S3

The application of **TZPzine-1** in detecting systems with different water samples. S4

NMR and HRMS data...S4

The comparison with typical probes for hydrazine..S6

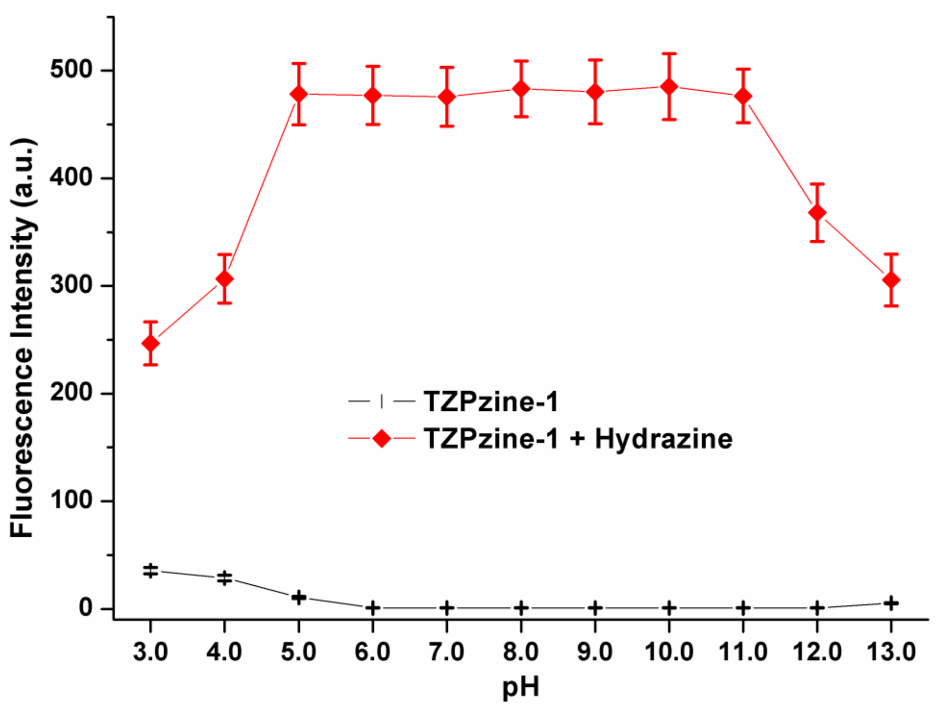

Figure S1. The fluorescence intensity at 460 nm changes in various pH environments. (Black: 10 μM **TZPzine-1**; Red: **TZPzine-1** with hydrazine). Temperature: 37 ℃; Incubation time: 20 min; Excitation wavelength: 365 nm.

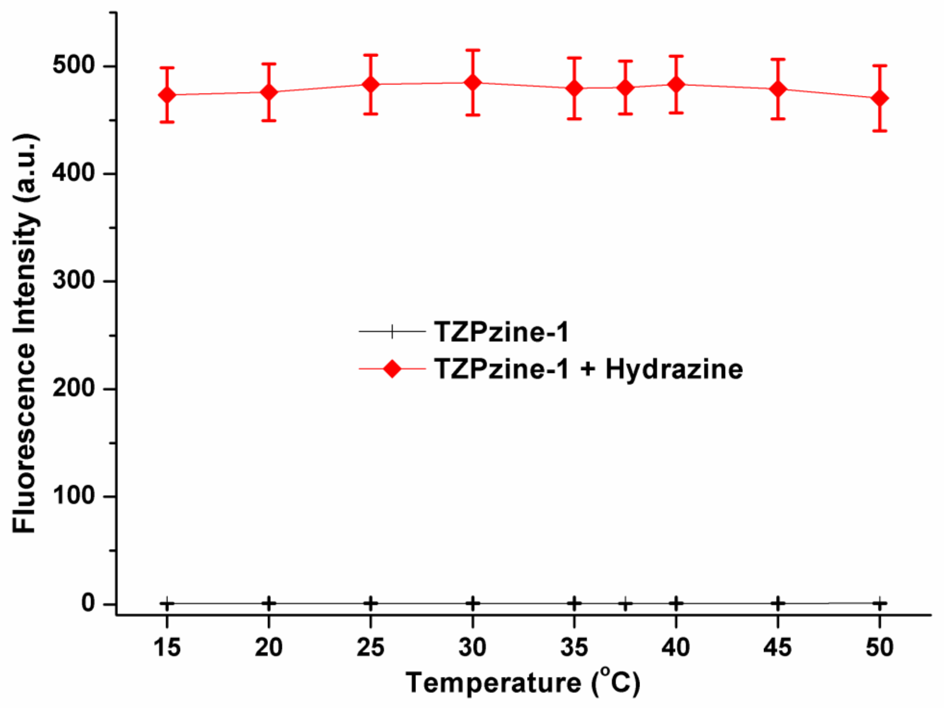

Figure S2. The fluorescence intensity at 460 nm changes in various temperature conditions. (Black: 10 μM **TZPzine-1**; Red: **TZPzine-1** with hydrazine). pH: 7.4; Incubation time: 20 min; Excitation wavelength: 365 nm.

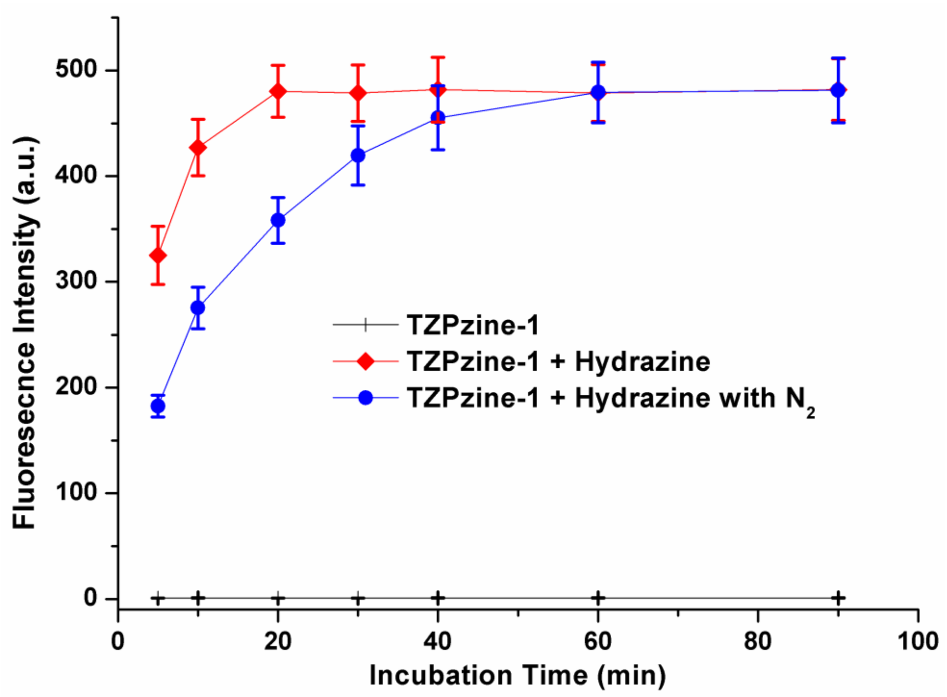

Figure S3. The fluorescence intensity at 460 nm changes in various time conditions. (Black: 10 μM **TZPzine-1**; Red: **TZPzine-1** with hydrazine; Blue: **TZPzine-1** with hydrazine with nitrogen). pH: 7.4; Temperature: 37 ℃; Excitation wavelength: 365 nm.

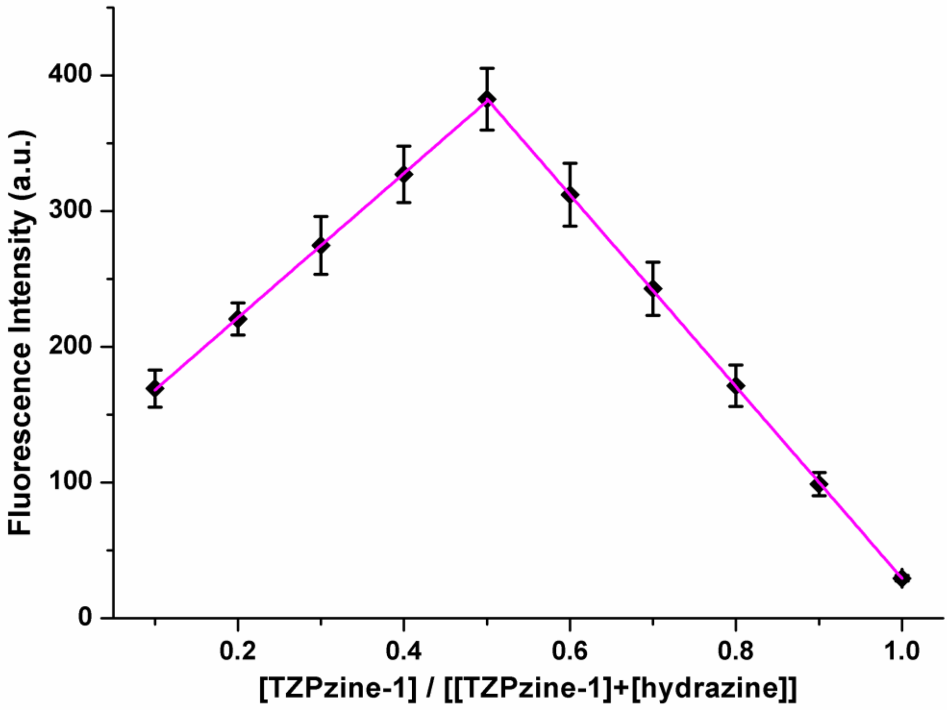

Figure S4. Job's plots analysis for **TZPzine-1** and hydrazine interaction with a total concentration of [**TZPzine-1**] + [hydrazine] at 200 μM by fluorescence response. The fluorescence emission at 460 nm was used.

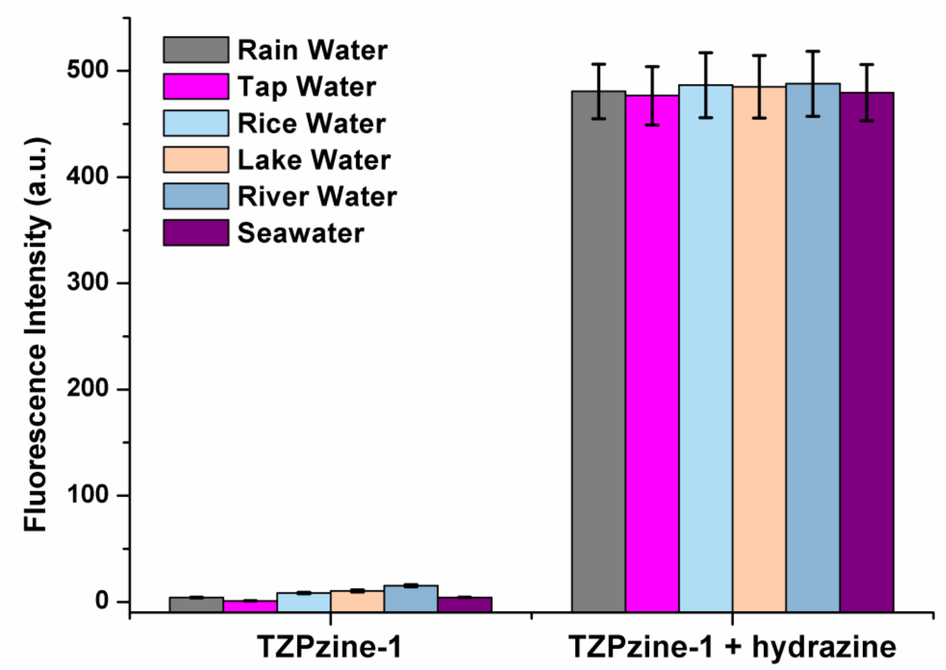

Figure S5. The application of **TZPzine-1** in detecting systems with different water samples from rain, tap, rice, lake, river and sea.

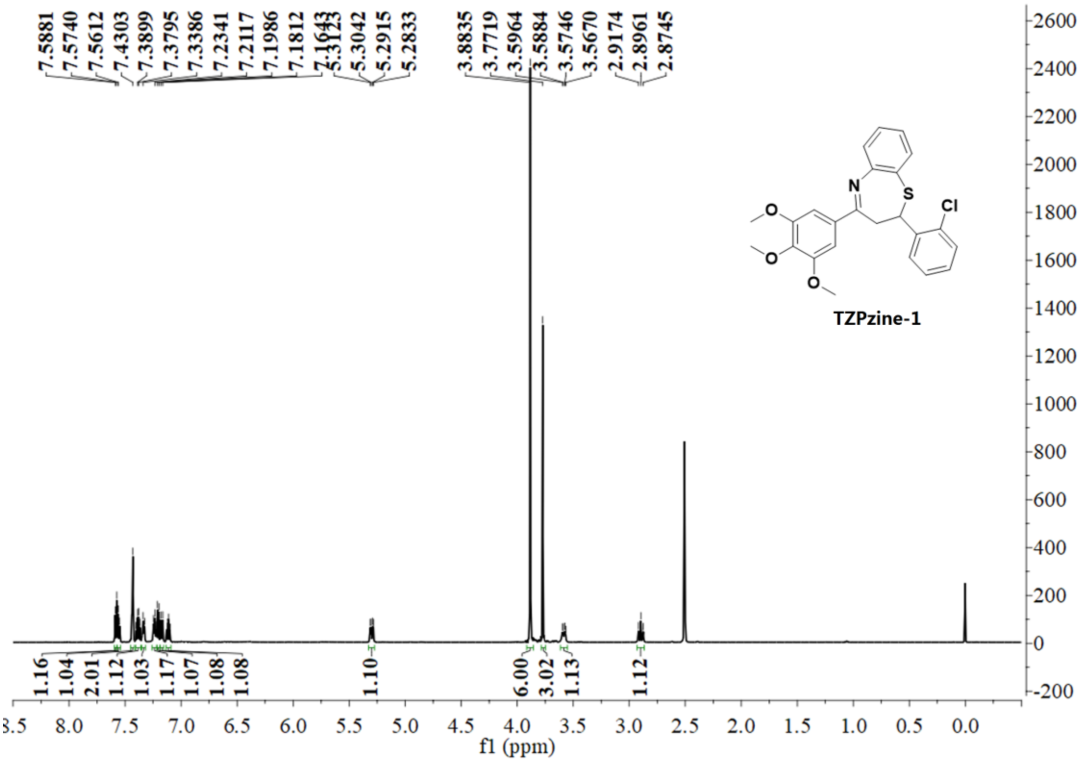

Figure S6. ^{1}H NMR of the probe **TZPzine-1** (600 MHz, in DMSO-d_6).

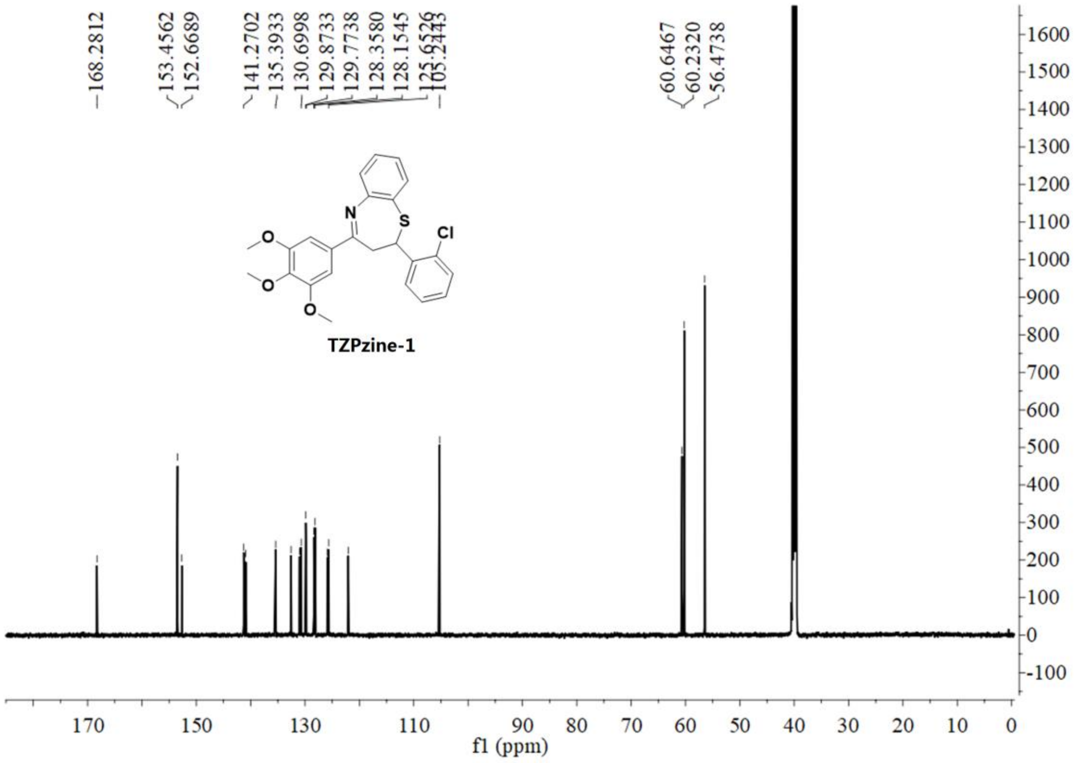

Figure S7. ^{13}C NMR of the probe **TZPzine-1** (151 MHz, in DMSO-d_6).

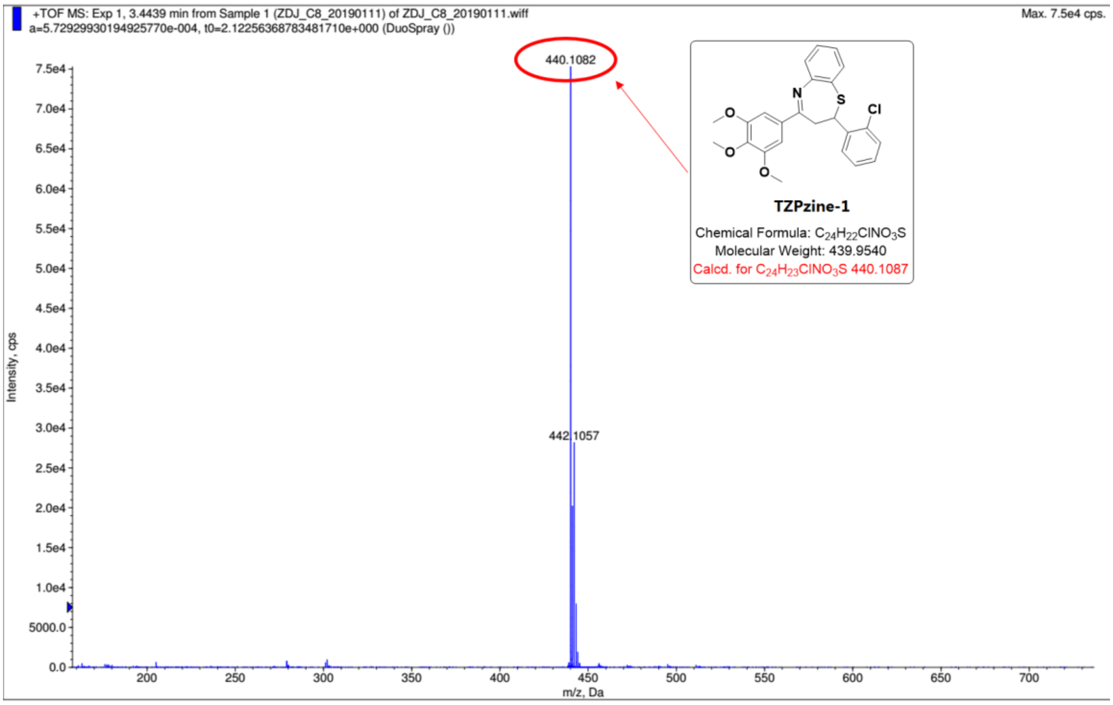

Figure S8. HRMS spectrum of the probe **TZPzine-1** in acetonitrile.

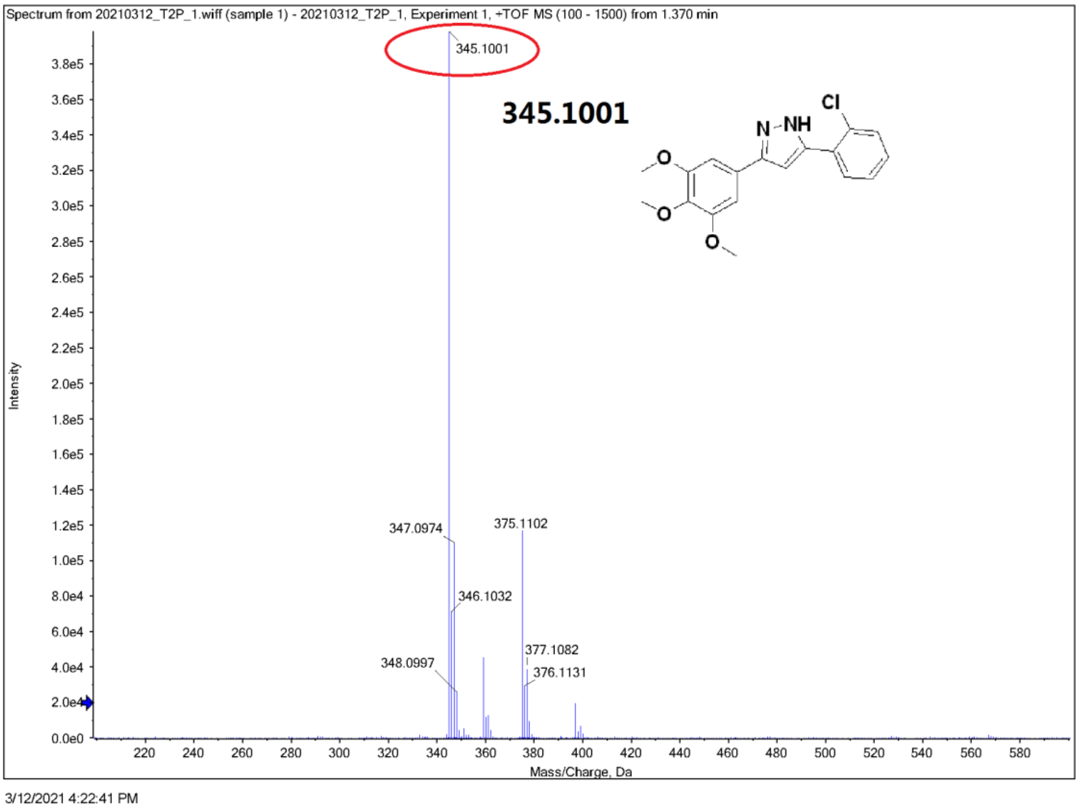

Figure S9. HRMS spectrum of the detecting product in acetonitrile.

Table S1. The comparison with typical probes for hydrazine.

Reference	Recognition Group	$\lambda_{em}/\lambda_{ex}$ (nm)	Response degree (-fold)	Test time (min)	LOD (nM)	Application
Ref 14	4-bromo butyrate	458/343<br>368/285[a]	88	1	66	*Candida albicans*
Ref 15	trifluoroacetyl acetonate	501/348<br>445/295[a]	33	60	100	TLC plate, HeLa cells
Ref 16	2-(pyridin-4-yl)acetonitrile	510/450<br>640/560[a]	3.3	60	380	TLC plate, A549 cells, Zebrafish
Ref 17	benzopyrylium	460/415<br>694/647[a]	8	30	87	TLC plate, HeLa cells
Ref 18	dinitrile	490/350	80	30	12.2	HeLa cells, Zebrafish
Ref 19	phthalimide	439/371	350	60	2673	Filter paper
Ref 20	4-bromo butyrate	627/580<br>814/780[a]	23	17	12	HeLa cells, Mice
Ref 21	acetyl	400/341<br>470/382[a]	10	7	31000	HeLa cells
Ref 22	levulinate	458/360	250	15	2460	None

Ref 23	4-bromo butyrate	521/360 387/360[a]	3050	30	5.8	Test paper, Water samples, MDA-MB-231 cells
Ref 24	phthalimide	430/360	60	40	48	Hct116 cells, HepG2 cells
Ref 25	aldehyde	556/391	38	60	1.12	Paper strip, Soil samples, Tissue
Ref 26	cyano	516/435 572/525[a]	> 100	12	163	Water samples, HeLa cells
Ref 27	acetyl	480/371	49	40	120	Water samples, MCF-7 cells, *Arabidopsis Thaliana*
**This work**	**benzo-thiazepine**	**460/365**	**480**	**20**	**50**	**Water samples, HeLa cells**

[a] The wavelength of the second emission and corresponding excitation (ratiometric analysis).
